# Peer Review of “Interpreting the Estimand Framework From a Causal Inference Perspective”

**DOI:** 10.2196/98125

**Published:** 2026-05-22

**Authors:** Qi Zhang

**Affiliations:** 1 Emory University School of Medicine, Atlanta, GA, United States

**Keywords:** causal inference, clinical trial, estimand, intercurrent event, treatment effect

*This is a peer review report for “Interpreting the Estimand Framework From a Causal Inference Perspective.”*

## Round 1 Review

### General Comments

This manuscript [1] aims to connect the International Council for Harmonisation of Technical Requirements for Pharmaceuticals for Human Use (ICH) E9 (R1) estimand framework with causal inference concepts; however, the exposition suffers from imprecise causal language and several conceptual inaccuracies. In particular, some core causal notions—such as potential outcomes, confounding in randomized trials, and the definition of the average treatment effect (ATE)—are stated incorrectly or inconsistently. In addition, established intercurrent-event strategies from ICH E9 (R1) are presented in a way that may suggest methodological novelty, despite being well known. Clarifying the causal assumptions, correcting the technical definitions, and more clearly distinguishing estimands from estimation methods would substantially improve the rigor and clarity of the manuscript.

### Specific Comments

#### Major Comments

1. The introduction may give the impression that these strategies are newly proposed by the author, whereas they are in fact defined in ICH E9 (R1). The manuscript would benefit from clearer attribution to, and positioning relative to, the ICH E9(R1) estimand framework.

2. The important concept of intercurrent events is not clearly defined. The definition provided in the manuscript, “Intercurrent events are events that happen after treatment initiation and affect the definition of a treatment effect” (page 2) is vague and potentially misleading. It misses the key idea that intercurrent events are posttreatment events that interfere with the interpretation or existence of the outcome relative to the treatment of interest, rather than merely events that affect treatment effects.

3. In section 2, it is incorrect to state that “Ri, Xi and Yi are potential outcomes.” Only Xi (⋅) and Yi (⋅) are potential outcomes. The randomization indicator Ri not a potential outcome; it is a realized random variable determined by the design.

4. At the beginning of section 2, the authors assume “an ideal two-arm randomized controlled clinical trial, with full compliance to treatment and no intercurrent events.” In such a setting, confounders do not affect treatment assignment. However, the manuscript later defines “some confounders C that affect both X and Y,” which contradicts the assumption of randomization.

5. ATE is defined as ATE = E(Y (X(*R* = 1) = 1) | C) − E(Y (X(*R* = 0) = 0) | C). However, this is a conditional ATE rather than the marginal ATE, since Y (X(*R* = 1) = 1) and Y (X(*R* = 0) = 0) are potential outcomes. The author should define the ATE marginally and then mention conditioning on C for adjustment.

6. Page 3: the phrase “the difference (D) between the average treatment effect from participants who take the experimental treatment...” is incorrect. The quantity described is the difference in average observed outcomes, not an average treatment effect.

7. Across strategies, the author repeatedly claims that the estimand formula is “still” the same, which is misleading. The symbolic form may look similar, but the estimand is not the same. In treatment policy, X is redefined; in composite and while-on-treatment strategies, Y is redefined; in principal stratification, the target population changes. This undermines the central E9 (R1) message that different strategies define different estimands.

8. The proposed “model adjustment strategy” does not correspond to an estimand strategy as defined in ICH E9 (R1), but rather to a particular modeling or estimation approach. Moreover, in case 1 of Figure 2, concomitant therapies occur after treatment initiation, which is inconsistent with the causal diagram in Figure 3. In this setting, M may act as a mediator rather than a confounder. Treating postrandomization intercurrent events as confounders requires careful causal justification and may induce bias; this issue is not discussed in the manuscript.

#### Minor Comments

9. Please spell out the abbreviation “ICH” at its first occurrence.

10. Some sentences are confusing and would benefit from revision. For example, in the second paragraph on page 2: “Intercurrent events are frequent in practice but conceptually novel. E9(R1) listed many examples for intercurrent events, such as use of concomitant therapies, treatment switching and death before endpoint measurement.” Intercurrent events are not really new conceptually; rather, they were newly formalized or explicitly emphasized in E9 (R1). In “examples for intercurrent events,” the preposition should be “of,” not “for.” As a second example, “This individual treatment effect controls confounders on the endpoint within the same participant and means how the endpoint would change when only the treatment condition changes” on page 3: the ITE does not “control confounders”; it is defined counterfactually for the same individual.

11. A right parenthesis is missing in the first ATE formula on page 3.

12. Equation 2.1 is missing the observed randomization indicator R^o in the first line.

13. The exclusion restriction assumption for instrumental variables should be stated more clearly.

## Abbreviations

ATE: average treatment effect
ICH: International Council for Harmonisation of Technical Requirements for Pharmaceuticals for Human Use
